# Differential Site-Based Expression of Pentose Phosphate Pathway-Related Proteins among Breast Cancer Metastases

**DOI:** 10.1155/2017/7062517

**Published:** 2017-02-02

**Authors:** Yoon Jin Cha, Woo Hee Jung, Ja Seung Koo

**Affiliations:** ^1^Department of Pathology, Gangnam Severance Hospital, Yonsei University College of Medicine, 211 Eonju-ro, Gangnam-gu, Seoul 06273, Republic of Korea; ^2^Department of Pathology, Severance Hospital, Yonsei University College of Medicine, 50-1 Yonsei-ro, Seodaemun-gu, Seoul 03722, Republic of Korea

## Abstract

*Purpose*. We aimed to investigate the expression of pentose phosphate pathway- (PPP-) related proteins in metastatic breast cancer and its relationship with clinicopathologic factors.* Methods*. Tissue samples from 126 metastatic breast cancers were included in a tissue microarray. Expression of PPP-related proteins [glucose-6-phosphate dehydrogenase (G6PDH), 6-phosphogluconolactonase (6PGL), 6-phosphogluconate dehydrogenase (6PGDH), and nuclear factor erythroid 2-related factor (NRF2)] was determined by immunohistochemistry.* Results*. G6PDH (*p* = 0.011) and cytoplasmic NRF2 (*p* = 0.001) showed the highest expression in brain metastases. Human epidermal growth factor receptor (HER-2) positivity was associated with G6PDH (*p* < 0.001) and cytoplasmic NRF2 (*p* = 0.015) positivity. A high Ki-67 labeling index (LI) was correlated with nuclear NRF2 positivity (*p* = 0.037), and HER-2-positive luminal B type was associated with G6PDH positivity (*p* = 0.001). On multivariate Cox analysis, independent risk factors of short overall survival were 6PGL positivity in bone metastasis (HR 4.180, 95% CI 1.160–15.06, *p* = 0.029) and low Ki-67 LI in lung metastasis (HR 11.853, 95% CI 1.841–76.30, *p* = 0.009).* Conclusion*. Differential expression of PPP-related proteins correlated with different prognoses and metastatic sites, with the highest expression in brain metastases, and could be a potential therapeutic target.

## 1. Introduction 

The pentose phosphate pathway (PPP) is a metabolic pathway parallel to glycolysis. The PPP links glucose metabolism with ribose production and NADPH generation. The PPP comprises oxidative and nonoxidative branches. The oxidative branch generates NADPH and ribonucleotides, with enzymatic regulation by glucose-6-phosphate dehydrogenase (G6PDH), 6-phosphogluconolactonase (6PGL), and 6-phosphogluconate dehydrogenase (6PGDH). Most of the pentose phosphate in the body, which is required in rapidly proliferative cells, is derived from the PPP. In cancer cells, the PPP generates pentose phosphate as well as NADPH, which is important in lipid synthesis and cell survival under stressful conditions. Thus, the PPP plays a pivotal role in constantly proliferating cancer cells, and increased expression of PPP-related enzymes in cancer tissue has been reported [1–3].

Breast cancer has high morbidity and mortality rates, caused by distant metastasis of primary tumors. Breast cancer commonly metastasizes to the lung, brain, liver, and bone [4, 5], and brain and bone metastases have been thoroughly investigated [6–11]. Tumor metastasis generally occurs by reciprocal interaction between tumor cells and host tissue via adhesion, proteolysis, invasion, and angiogenesis [4, 12]. Because not all tumors have similar metastatic patterns, the seed and soil hypothesis was proposed to explain tumor metastasis as the survival of a specific tumor (seed) in a specific visceral organ (soil) [13]. Breast cancer metastases have different signatures according to the metastatic sites. Brain metastases have specific clinical characteristics such as young patient age, estrogen receptor (ER) negativity, prior lung metastasis, human epidermal growth factor receptor- (HER-) 2 amplification, epidermal growth factor receptor (EGFR) overexpression, and basal subtype [8, 9, 11]. In contrast, bone metastases are correlated with low histologic grade, ER positivity, ER positivity/progesterone receptor (PR) negativity, strand growth pattern, and the presence of fibrotic tumor stroma [7, 14, 15]. Therefore, it is expected that different metastatic sites would show different expression patterns of PPP-related proteins; however, this has not been well studied.

In the present study, we aimed to analyze the expression of PPP-related proteins at different metastatic sites of metastatic breast cancer and to identify the relationship between protein expression and clinicopathologic factors.

## 2. Materials and Methods

This study was approved by the Institutional Review Board of Severance Hospital.

### 2.1. Patient Selection

Invasive primary breast cancers and their metastases to distant organs (liver, lung, brain, and bone) were retrieved from the data files of the Department of Pathology of Severance Hospital. Only patients with a diagnosis of invasive ductal carcinoma were included. A total of 162 cases were selected with 49 pairs of primary tumors and their metastases. All slides were re-reviewed and pathologic diagnoses were approved by two pathologists (JSK and WJ). The histological grade was assessed using the Nottingham grading system [16].

### 2.2. Tissue Microarray

Hematoxylin and eosin- (H&E-) stained tumor samples were mounted on slides, a representative area was selected, and a corresponding spot was marked on the surface of the paraffin block. Using a biopsy needle, the selected area was punched out and a 3 mm tissue core was placed onto a 6 × 5 recipient block. Tissue was extracted from invasive tumors. More than two tissue cores were extracted to minimize extraction bias. Each tissue core was assigned a unique tissue microarray location number that was linked to a database containing other clinicopathologic data.

### 2.3. Immunohistochemistry

The antibodies used for immunohistochemistry (IHC) in this study are listed in Table 1. IHC was performed with formalin-fixed, paraffin-embedded tissue sections. Briefly, 3 *μ*m thick tissue sections from paraffin blocks were deparaffinized, rehydrated with xylene and alcohol solution, and stained using Ventana Discovery XT automated stainer (Ventana Medical System, Tucson, AZ, USA). CC1 buffer (Cell Conditioning 1; citrate buffer pH 6.0, Ventana Medical System) was used for antigen retrieval. Appropriate positive and negative controls were included.

### 2.4. Interpretation of Immunohistochemical Results

A cut-off value of 1% or more positively stained nuclei was used to define ER and PR positivity [17]. HER-2 staining was analyzed according to the American Society of Clinical Oncology/College of American Pathologists guidelines using the following categories: 0 = no immunostaining; 1+ = weak incomplete membranous staining in less than 10% of tumor cells; 2+ = complete membranous staining that is either uniform or weak in at least 10% of tumor cells; and 3+ = uniform intense membranous staining in at least 30% of tumor cells [18]. HER-2 immunostaining was considered positive when strong (3+) membranous staining was observed and was considered negative for tumors with 0 to 1+ staining. The tumors showing 2+ HER-2 expression were further evaluated for HER-2 amplification by using silver in situ hybridization (SISH).

IHC results were interpreted after multiplying the staining intensity score (negative, 0; weak, 1; moderate, 2; strong, 3) and the proportion of stained cells (negative, 0; <30% stained, 1; ≥30% stained, 2). Scores of 0 and 1 were considered negative, and scores of 2–4 and 5-6 were considered low and high positivity, respectively [19]. The Ki-67 labeling index (LI) was defined as the percentage of nuclear-stained tumor cells.

### 2.5. Tumor Phenotype Classification

We classified breast cancer phenotypes according to the IHC results for ER, PR, HER-2 and Ki-67, and SISH results for HER-2 as follows [20]:* luminal A type*: ER or/and PR positive and HER-2 negative and Ki-67 LI <14%;* luminal B type*: (HER-2 negative) ER or/and PR positive and HER-2 negative and Ki-67 LI ≥14% and (HER-2 positive) ER or/and PR positive and HER-2 overexpressed or/and amplified;* HER-2 type*: ER and PR negative and HER-2 overexpressed or/and amplified; and* triple negative breast cancer* (*TNBC) type*: ER, PR, and HER-2 negative.

### 2.6. Statistical Analysis

Data were statistically analyzed using SPSS for Windows, version 12.0 (SPSS Inc., Chicago, IL, USA). Correlation analysis of immunostaining results between primary breast cancer and metastatic breast cancer was performed using the McNemar test. Student's *t*-test and Fisher's exact test were used to examine any differences in continuous and categorical variables, respectively. A corrected *p* value and the Bonferroni method were used for multiple comparisons. Statistical significance was assumed when *p* < 0.05. Kaplan-Meier survival curves and log-rank statistics were employed to evaluate time to tumor metastasis and time to survival. Multivariate regression analysis was performed using a Cox proportional hazards model.

## 3. Results

### 3.1. Clinicopathologic Characteristics of Metastatic Breast Cancer

A total of 126 metastatic breast cancers comprised 31 (24.6%) bone metastases, 36 (28.6%) brain metastases, 11 (8.7%) liver metastases, and 48 (38.1%) lung metastases (Table 2). ER (*p* < 0.001), PR (*p* < 0.001), HER-2 (*p* = 0.032), Ki-67 LI (*p* = 0.008), and molecular subtype (*p* < 0.001) differed with regard to the metastatic sites. ER negativity, PR negativity, and HER-2 positivity were more frequent and Ki-67 LI was higher for brain metastases than for the other sites. Predominant molecular subtype was luminal A in bone and liver metastases, HER-2 in brain metastases, and TNBC in lung metastases.

### 3.2. Expression of Pentose Phosphate Pathway-Related Proteins in Metastatic Breast Cancer

**Figure 1:**
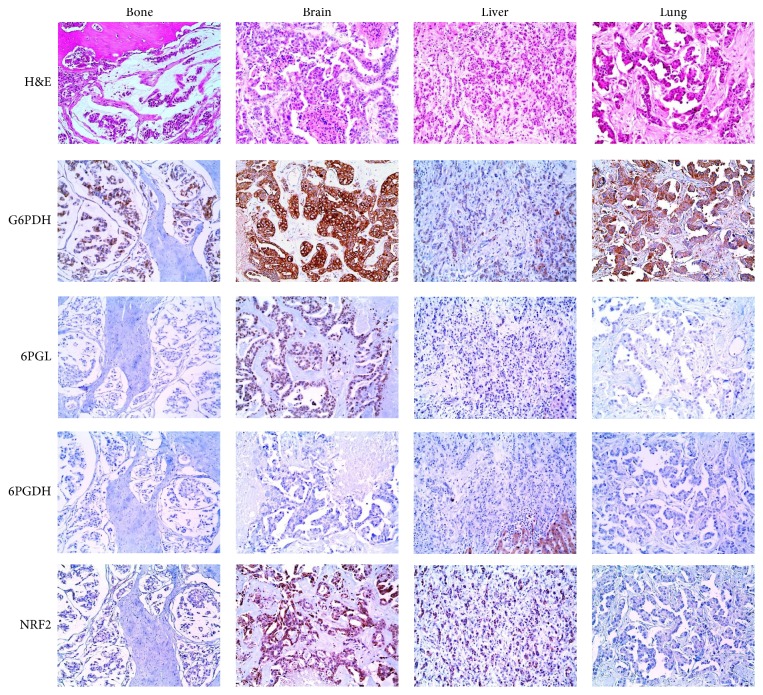
Expression of pentose phosphate pathway-related proteins in metastatic breast cancer. The expression of G6PDH and cytoplasmic NRF2 in brain metastases is higher than that at other sites.

**Figure 2:**
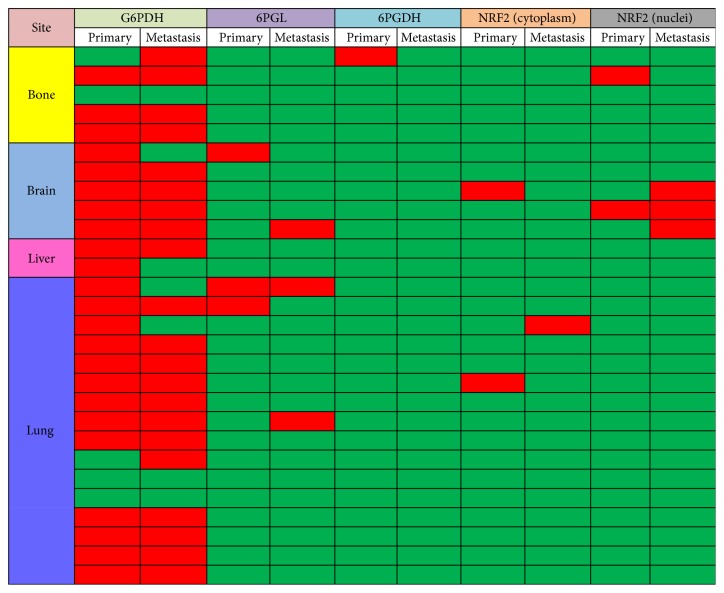
Expression status of pentose phosphate pathway-related proteins in paired primary and metastatic breast cancer (red, positive; green, negative).

G6PDH (*p* = 0.011) and cytoplasmic NRF2 (*p* = 0.001) in metastatic breast cancers were differentially expressed depending on the metastatic sites, with brain metastases showing higher expression of G6PDH and cytoplasmic NRF2 than the other sites (Figure 1 and Table 3). Comparisons of the 28 paired primary metastatic breast cancers revealed differential expression patterns of G6PDH (*n* = 6, 21.4%, *p* = 0.688), 6PGL (*n* = 4, 14.3%, *p* = 1.000), 6PGDH (*n* = 1, 3.6%, *p* = 1.000), cytoplasmic NRF2 (*n* = 3, 10.7%, *p* = 1.000), and nuclear NRF2 (*n* = 3, 10.7%, *p* = 1.000) (Figure 2). Expression rates of 6PGL, 6PGDH, and cytoplasmic NRF2 were relatively low in metastatic breast cancer and primary breast cancer; the mentioned rates were 29.4%, 3.2%, and 10.3% in metastatic breast cancer and 10.7%, 3.6%, and 7.1% in primary breast cancer, respectively.

### 3.3. Correlation between Clinicopathologic Factors and Expression of Pentose Phosphate Pathway-Related Proteins

**Figure 3:**
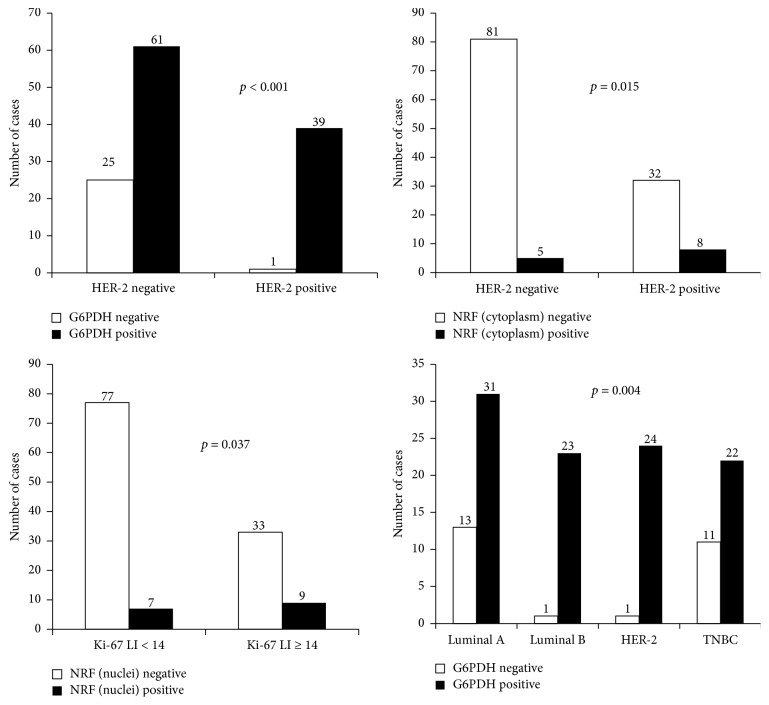
Correlation between clinicopathologic factors and expression of pentose phosphate pathway-related proteins.

HER-2 amplification was associated with G6PDH positivity (*p* < 0.001) and cytoplasmic NRF2 positivity (*p* = 0.015). Higher Ki-67 LI was correlated with higher nuclear NRF2 expression (*p* = 0.037). Luminal B (HER-2 positive) type was associated with G6PDH positivity (*p* = 0.001) (Figure 3).

### 3.4. The Impact of Expression of Pentose Phosphate Pathway-Related Proteins on Prognosis in Metastatic Breast Cancer

**Figure 4:**
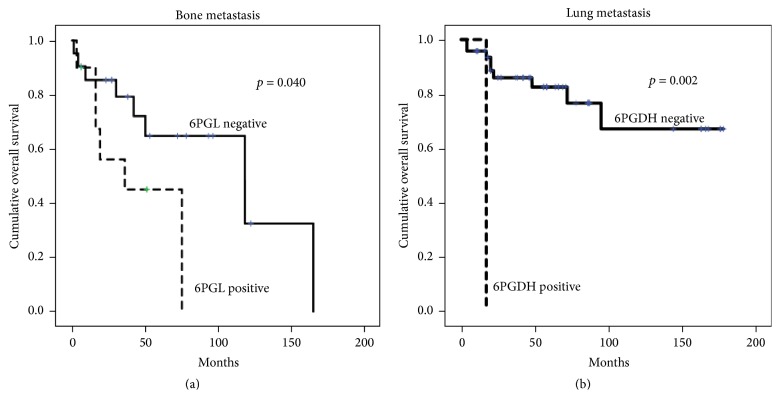
Overall survival according to the expression of pentose phosphate pathway-related proteins in bone metastases (a) and lung metastases (b). Shorter overall survival is associated with 6PGL positivity in bone metastases and 6PGDH positivity in lung metastases.

Univariate analysis of all metastatic breast cancer cases revealed that the expression of PPP-related proteins had no effect on patient prognosis (Table 4). However, in terms of metastatic sites, expression of 6PGL in bone metastases and 6PGDH in lung metastases was associated with shorter overall survival (*p* = 0.040 and *p* = 0.002, resp., Figure 4). On multivariate Cox analysis, 6PGL positivity (hazard ratio [HR] 4.180; 95% confidential interval [CI] 1.160–15.06; *p* = 0.029) and lower Ki-67 LI (HR 11.853; 95% CI 1.841–76.30; *p* = 0.009) were independent risk factors for shorter overall survival in bone metastasis and lung metastasis, respectively (Table 5).

## 4. Discussion

We investigated the expression of PPP-related proteins in metastatic breast cancers and observed differential expression patterns depending on the metastatic sites. Brain metastases showed higher expression of G6PDH and cytoplasmic NRF2. The site-based variations in the cell biology of metastatic tumors could result in the differential expression of PPP-related proteins at each metastatic site. In the present study, we found that HER-2 positivity correlated with G6PDH and cytoplasmic NRF2 expression. A previous study in an ErbB2-positive breast cancer cell line BT-474 revealed that knockdown of NRF2 inhibited HER-2 expression [21]. NRF2 is key molecule in the regulation of the PPP and also regulates PPP-related protein expression in tumors [22], which would be affected by specific tumor cells types. Another potential mechanism for differential expression of PPP-related proteins is tumor microenvironment. Various tumor environments could influence the PPP. Compared to the PPP in healthy tissue, the PPP flux is higher in traumatically injured brain tissue [23, 24], as well as in brain tumors because of the involvement of NRF2 [22]; thus, an increase in PPP activity is possible in brain metastasis. NRF2 is a nuclear transcription factor that contributes to cellular differentiation, proliferation, and inflammation and that is involved in antioxidant gene activity in neurodegeneration [25] and cardiovascular disease [26]. In human cancers, overexpression of nuclear NRF2 is associated with tumor progression and drug resistance [27, 28], and a correlation between nuclear NRF2 expression and higher Ki-67 LI was observed in the present study. Moreover, we found that cytoplasmic NRF2 expression was correlated with HER-2 positivity, mostly in brain metastases. Cytoplasmic expression of NRF2 represents aberrant subcellular localization. In colorectal cancer, cytoplasmic NRF2 expression has been reported to promote cancer cell invasion via regulation of PSMD4 [29], and a higher frequency of cytoplasmic NRF2 in HER-2-positive cancers and brain metastases might reflect greater invasiveness and aggressiveness.

We found that shorter overall survival was associated with 6PGL positivity in bone metastases and 6PGDH positivity in lung metastases. Expression of PPP-related proteins is associated with poor prognosis in esophageal cancer [30], colon cancer [31], and ocular adnexal tumor [32]. These findings are consistent with our results, suggesting that PPP-related proteins could be prognostic factors in patients with metastatic breast cancer, especially in patients with bone metastasis. However, further study is required to validate our findings before their application in clinical practice. The results of the present study indicate that PPP-related proteins could be a potential therapeutic target in metastatic breast cancer, particularly for brain metastases, which had a higher expression of PPP-related proteins. In previous studies, inhibition of PPP-related proteins induced growth suppression and cell death in leukemia [33], ovary cancer [34], urinary bladder cancer [35], and breast and prostate cancer [36], which suggested that control of expression of PPP-related proteins could be an effective treatment strategy. Therefore, further development of PPP-related protein targeting agent should be evaluated in metastatic breast cancer patients through clinical trials. In conclusion, PPP-related proteins in metastatic breast cancer showed different expression patterns that were specific to the metastatic sites, with increased expression in brain metastases. Expression of PPP-related proteins at specific metastatic sites correlated with prognosis.

## Figures and Tables

**Table 1:** Source, clone, and dilution of antibodies.

Antibody	Company	Clone	Dilution
*Pentose phosphate pathway*-*related proteins*			
G6PDH	Abcam, Cambridge, UK	Polyclonal	1 : 100
6PGL	Abcam, Cambridge, UK	ERP1238(B)	1 : 200
6PGDH	Abcam, Cambridge, UK	Polyclonal	1 : 100
NRF2	Abcam, Cambridge, UK	Polyclonal	1 : 50
*Molecular subtype related proteins*			
ER	Thermo Scientific, San Diego, CA, USA	SP1	1 : 100
PR	DAKO, Glostrup, Denmark	PgR	1 : 50
HER-2	DAKO, Glostrup, Denmark	Polyclonal	1 : 1500
Ki-67	Abcam, Cambridge, UK	MIB	1 : 1000

G6PDH, glucose-6-phosphate dehydrogenase; 6PGL, 6-phosphogluconolactonase; 6PGDH, 6-phosphogluconate dehydrogenase; NRF2, nuclear factor erythroid 2- (NF-E2-) related factor 2; ER, estrogen receptor; PR, progesterone receptor; HER-2, human epidermal growth factor-2.

**Table 2:** Basal characteristics of patients with metastatic breast cancer.

Parameter	Total *N* = 126 (%)	Metastatic site				*p* value
		Bone *N* = 31 (%)	Brain *N* = 36 (%)	Liver *N* = 11 (%)	Lung *N* = 48 (%)	
Age (years)						
≤50	65 (51.6)	17 (54.8)	17 (47.2)	4 (36.4)	27 (56.3)	**0.605**
>50	61 (48.4)	14 (45.2)	19 (52.8)	7 (63.6)	21 (43.8)	
ER						
Negative	59 (46.8)	6 (19.4)	25 (69.4)	2 (18.2)	26 (54.2)	**<0.001**
Positive	67 (53.2)	25 (80.6)	11 (30.6)	9 (81.8)	22 (45.8)	
PR						
Negative	86 (68.3)	16 (51.6)	35 (97.2)	3 (27.3)	32 (66.7)	**<0.001**
Positive	40 (31.7)	15 (48.4)	1 (2.8)	8 (72.7)	16 (33.3)	
HER-2						
Negative	86 (68.3)	25 (80.6)	18 (50.0)	9 (81.8)	34 (70.8)	**0.032**
Positive	40 (31.7)	6 (19.4)	18 (50.0)	2 (18.2)	14 (29.2)	
Ki-67 LI						
<14	84 (66.7)	27 (87.1)	18 (50.0)	9 (81.8)	30 (62.5)	**0.008**
≥14	42 (33.3)	4 (12.9)	18 (50.0)	2 (18.2)	18 (37.5)	
Molecular subtype						
Luminal A	44 (34.9)	21 (67.7)	3 (8.3)	6 (54.5)	14 (29.2)	**<0.001**
Luminal B	24 (19.0)	5 (16.1)	8 (22.2)	3 (27.3)	8 (16.7)	
HER-2	25 (19.8)	3 (9.7)	12 (33.3)	1 (9.1)	9 (18.8)	
TNBC	33 (26.2)	2 (6.5)	13 (36.1)	1 (9.1)	17 (35.4)	
Patient death	41 (32.5)	16 (51.6)	11 (30.6)	4 (36.4)	10 (20.8)	**0.041**

ER, estrogen receptor; PR, progesterone receptor; HER-2, human epidermal growth factor-2; LI, labeling index; TNBC, triple negative breast cancer.

**Table 3:** Expression of pentose phosphate pathway-related proteins according to the metastatic site in breast cancer metastases.

Parameter	Total *N* = 126 (%)	Metastatic site				*p* value
		Bone *N* = 31 (%)	Brain *N* = 36 (%)	Liver *N* = 11 (%)	Lung *N* = 48 (%)	
G6PDH						
Negative	26 (20.6)	7 (22.6)	3 (8.3)	6 (54.5)	10 (20.8)	**0.011**
Positive	100 (79.4)	24 (77.4)	33 (91.7)	5 (45.5)	38 (79.2)	
6PGL						
Negative	89 (70.6)	21 (67.7)	23 (63.9)	11 (100.0)	34 (70.8)	0.139
Positive	37 (29.4)	10 (32.3)	13 (36.1)	0 (0.0)	14 (29.2)	
6PGDH						
Negative	122 (96.8)	30 (96.8)	34 (94.4)	11 (100.0)	47 (97.9)	0.750
Positive	4 (3.2)	1 (3.2)	2 (5.6)	0 (0.0)	1 (2.1)	
NRF2 (cytoplasm)						
Negative	113 (89.7)	31 (100.0)	26 (72.2)	11 (100.0)	45 (93.8)	**0.001**
Positive	13 (10.3)	0 (0.0)	10 (27.8)	0 (0.0)	3 (6.3)	
NRF2 (nuclei)						
Negative	110 (87.3)	28 (90.3)	28 (77.8)	11 (100.0)	43 (89.6)	0.170
Positive	16 (12.7)	3 (9.7)	8 (22.2)	0 (0.0)	5 (10.4)	

G6PDH, glucose-6-phosphate dehydrogenase; 6PGL, 6-phosphogluconolactonase; 6PGDH, 6-phosphogluconate dehydrogenase; NRF2, nuclear factor erythroid 2- (NF-E2-) related factor 2.

**Table 4:** Univariate analysis of the association between expression levels of pentose phosphate pathway-related proteins in metastatic breast cancers and overall survival by the log-rank test.

Parameters	Total, *N* = 149 (%)		Metastatic site							
			Bone, *N* = 31 (%)		Brain, *N* = 36 (%)		Liver, *N* = 11 (%)		Lung, *N* = 48 (%)	
	Mean survival	*p* value	Mean survival	*p* value	Mean survival	*p* value	Mean survival	*p* value	Mean survival	*p* value
	(95% CI) months		(95% CI) months		(95% CI) months		(95% CI) months		(95% CI) months	
G6PDH										
Negative	109 (79–138)	0.732	65 (50–79)	0.390	24 (20–28)	0.534	80 (47–112)	0.761	102 (53–150)	0.064
Positive	116 (99–132)		79 (51–108)		106 (82–129)		60 (28–92)		142 (116–168)	
6PGL										
Negative	116 (99–133)	0.599	101 (68–133)	**0.040**	111 (83–139)	0.716	n/a	n/a	n/a	n/a
Positive	94 (62–127)		43 (23–64)		65 (44–87)		n/a		n/a	
6PGDH										
Negative	114 (98–129)	0.976	n/a	n/a	n/a	n/a	n/a	n/a	136 (111–160)	**0.002**
Positive	71 (27–115)		n/a		n/a		n/a		17 (17–17)	
NRF2 (cytoplasm)										
Negative	108 (92–125)	0.079	n/a	n/a	90 (61–119)	0.123	n/a	n/a	n/a	n/a
Positive	108 (91–126)		n/a		105 (82–128)		n/a		n/a	
NRF2 (nuclei)										
Negative	115 (99–131)	0.964	91 (62–120)	0.340	100 (74–127)	0.573	n/a	n/a	n/a	n/a
Positive	71 (55–86)		62 (36–87)		79 (56–102)		n/a		n/a	

CI, confidential interval; G6PDH, glucose-6-phosphate dehydrogenase; 6PGL, 6-phosphogluconolactonase; n/a, not applicable; 6PGDH, 6-phosphogluconate dehydrogenase; NRF2, nuclear factor erythroid 2- (NF-E2-) related factor 2.

**Table 5:** Multivariate Cox analysis of the association between expression levels of pentose phosphate pathway-related proteins in metastatic breast cancers and overall survival.

Included parameters	Bone metastasis			Lung metastasis		
	Overall survival			Overall survival		
	HR	95% CI	*p* value	HR	95% CI	*p* value
ER status						
Negative versus positive	1.768	0.113–27.61	0.685	n/a	n/a	n/a
PR status						
Negative versus positive	0.453	0.119–1.730	0.247	n/a	n/a	n/a
HER-2 status						
Negative versus positive	1.025	0.217–4.833	0.975	n/a	n/a	n/a
Ki-67 LI						
≤14 versus >14	0.961	0.031–29.98	0.982	11.853	1.841–76.30	**0.009**
Molecular subtype						
TNBC versus non-TNBC	0.647	0.002–254.8	0.886	n/a	n/a	n/a
6PGL						
Negative versus positive	4.180	1.160–15.06	**0.029**	n/a	n/a	n/a
6PGDH						
Negative versus positive	n/a	n/a	n/a	1.362	0.130–14.29	0.797

HR, hazard ratio; CI, confidential interval; ER, estrogen receptor; n/a, not applicable; PR, progesterone receptor; HER-2, human epidermal growth factor-2; LI, labeling index; TNBC, triple negative breast cancer; 6PGL, 6-phosphogluconolactonase; n/a, not applicable; 6PGDH, 6-phosphogluconate dehydrogenase.
